# Vision for action: thalamic and cortical inputs to the macaque superior parietal lobule

**DOI:** 10.1007/s00429-021-02377-7

**Published:** 2021-09-15

**Authors:** Michela Gamberini, Lauretta Passarelli, Matteo Filippini, Patrizia Fattori, Claudio Galletti

**Affiliations:** Department of Biomedical and Neuromotor Sciences, University of Bologna, 40126 Bologna, Italy

**Keywords:** Dorsal visual stream, Sensorimotor integration, Goal-directed arm movement, Area V6, Area V6A, Area PEc

## Abstract

The dorsal visual stream, the cortical circuit that in the primate brain is mainly dedicated to the visual control of actions, is split into two routes, a lateral and a medial one, both involved in coding different aspects of sensorimotor control of actions. The lateral route, named “lateral grasping network”, is mainly involved in the control of the distal part of prehension, namely grasping and manipulation. The medial route, named “reach-to-grasp network”, is involved in the control of the full deployment of prehension act, from the direction of arm movement to the shaping of the hand according to the object to be grasped. In macaque monkeys, the reach-to-grasp network (the target of this review) includes areas of the superior parietal lobule (SPL) that hosts visual and somatosensory neurons well suited to control goal-directed limb movements toward stationary as well as moving objects. After a brief summary of the neuronal functional properties of these areas, we will analyze their cortical and thalamic inputs thanks to retrograde neuronal tracers separately injected into the SPL areas V6, V6A, PEc, and PE. These areas receive visual and somatosensory information distributed in a caudorostral, visuosomatic trend, and some of them are directly connected with the dorsal premotor cortex. This review is particularly focused on the origin and type of visual information reaching the SPL, and on the functional role this information can play in guiding limb interaction with objects in structured and dynamic environments.

## Introduction

The parietal lobe of primates takes part in superior cognitive functions (attention, memory, language, executive functions; Vallar and Coslett 2018) that allow us to understand and to effectively interact with the world. The posterior part of the parietal lobe (the posterior parietal cortex that includes the superior and inferior parietal lobules) is largely expanded in primates and is involved in cognitive and perceptive abilities useful to guide searching behavior (Kaas et al. 2018; Rizzolatti et al. 2020). For a long time, the macaque superior parietal lobule (SPL), the main target of this review, has been considered a somatic structure, where the body, in particular the limbs, and particularly the upper limbs, are represented (Graziano et al. 2000; Sereno and Huang 2014; Gamberini et al. 2018). Recent research, however, has shown that also the visual input reaches the SPL, and today it is clear that visual and somatic inputs interact in the SPL allowing the guidance of reaching and grasping behavior. The analysis of this interaction, and in particular the role of vision in this process, is the goal of the present review.

**Fig. 1:**
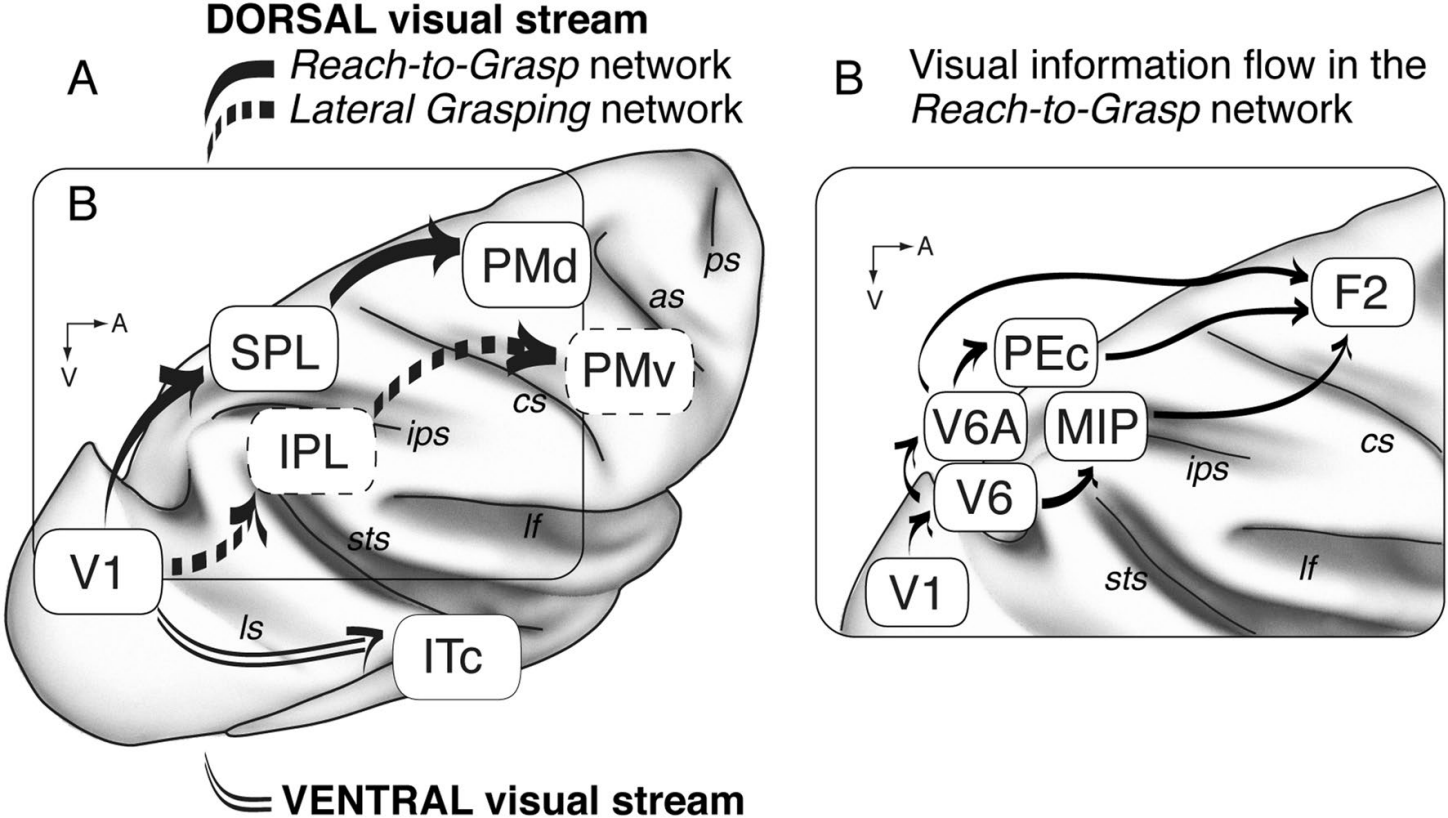
Subdivisions within the visual pathway from V1. Dorsal vs ventral visual streams and reach-to-grasp vs lateral grasping networks. **A** The dorsal visual stream is organized into two main routes: in the "reach-to-grasp" network (continuous thick arrows), visual information from V1 involves parietal areas of the superior parietal lobule (SPL) and reaches the dorsal premotor areas (PMd) (Fattori et al. 2017); the "lateral grasping" network (dashed arrows) involves parietal areas of the inferior parietal lobule (IPL) and reaches the ventral premotor areas (PMv) (Borra et al. 2017). In the ventral visual stream (double continuous thin arrows), the visual information from V1 reaches the inferior temporal cortex (ITc). **B** In the "reach-to-grasp" network, visual information, starting from V1, involves areas V6, V6A, PEc and MIP and reaches the premotor area F2. *cs* central sulcus, *as* arcuate sulcus, *ips* intraparietal sulcus, *lf* lateral fissure, *ps* principal sulcus, *sts* superior temporal sulcus, *ls* lunate sulcus, *pos* parieto-occipital sulcus, *V1, V6, V6A, PEc, MIP, F2* areas V1, V6, V6A, PEc, MIP, F2, *SPL* superior parietal lobule, IPL inferior parietal lobule, *ITc* inferior temporal cortex, *PMd* dorsal premotor cortex, *PMv* ventral premotor cortex, *A* anterior, *V* ventral

According to the well-known model proposed by Ungerleider and Mishkin (1982) visual information from the primary visual cortex (V1) reaches the extrastriate visual areas following two streams: a dorsal one (called "dorsal visual stream") that reaches the posterior parietal cortex and is involved in object location in space; and a ventral one (called "ventral visual stream") that reaches the inferior temporal cortex and is involved in object recognition (Fig. 1A). About 10 years later, Ungerleider and Mishkin proposed that the dorsal visual stream was involved in 'action' while the ventral one in 'perception' (Goodale and Milner 1992; Milner and Goodale 1995). According to this view, visual information in the dorsal visual stream would be used to prepare and control visually guided actions, while in the ventral visual stream it would be used to perceive and recognize objects presented in the visual field. Originally, visual information in the dorsal stream was thought to reach only the inferior part of the parietal lobe (the inferior parietal lobule, IPL; Ungerleider and Mishkin 1982), but later on it became evident that visual information reached also the SPL (Fig. 1A) (Milner and Goodale 1995; Jeannerod et al. 1995; Wise et al. 1997). Considering this evidence, the dorsal visual stream was split into two routes, a lateral and a medial one, both involved in encoding different aspects of sensorimotor control of actions (Galletti et al. 2003; Rizzolatti and Matelli 2003). The lateral route of the dorsal stream involves the IPL and ends in the ventral premotor cortex. It is mainly involved in the control of grasping and manipulation under perceptual and cognitive control (Borra et al. 2017) and has been recently named "lateral grasping network". The medial route of the dorsal stream, involving the SPL and ending in the dorsal premotor cortex, was originally described as a network involved only in the control of arm transport during reaching movements (Rizzolatti and Matelli 2003). More recently, however, it has been reported that the medial route of the dorsal stream is involved in the control of the entire sequence of acts during prehension, from the direction of arm movement to the shaping of the hand according to the object to be grasped (Fattori et al. 2004, 2005, 2012) and has been accordingly named “reach-to-grasp network” (Fattori et al. 2017; Galletti and Fattori 2018).

In this review, we will first summarize the functional properties of SPL areas, in particular the visual properties. Then, we will analyze the flow of visual information reaching the SPL (Fig. 1B). Cortical and subcortical inputs to the SPL will be analyzed taking into account the more recent areal subdivision of this part of the parietal cortex.

## Sensory properties of the superior parietal lobule

**Fig. 2:**
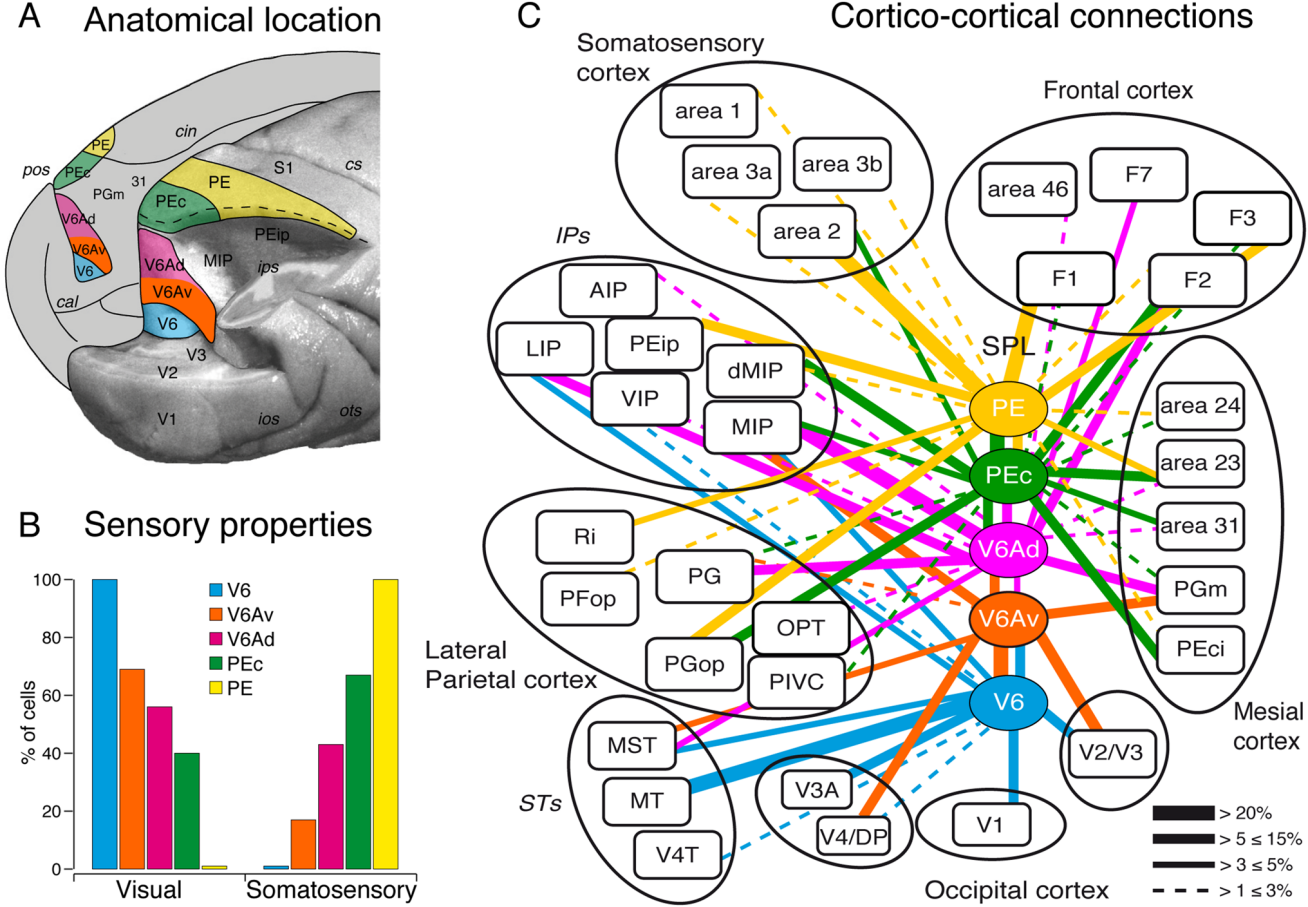
Anatomical location, sensory properties and corticocortical connections of the SPL areas. **A** Posterior view of macaque occipital and parietal lobes. The right hemisphere (posterolateral view) has been partially dissected at the level of the fundus of intraparietal, parieto-occipital, and lunate sulci to show the hidden cortex of SPL. The medial surface of the left hemisphere is also visible. Continuous lines delimit the different SPL areas (in color) described in this work. **B** Incidence of visual and somatosensory cells in areas V6, V6A (V6Av and V6Ad), PEc, and PE. Data are obtained by the following studies (Galletti et al. 1999a; Gamberini et al. 2011, 2018; De Vitis et al. 2019). **C** Summary of cortical connections of areas V6, V6A (V6Av and V6Ad), PEc and PE modified from the following studies (Galletti et al. 2001; Gamberini et al. 2009; Bakola et al. 2010, 2013; Passarelli et al. 2011). The boxes representing different areas are organized approximately in a caudal to rostral sequence, from the bottom part of the figure to the top. The proportion of neurons forming each connection is indicated by the thickness of the bars linking different areas. *cal* calcarine fissure, *cin* cingulate sulcus, *V2, V3, V3A, V4/DP, V4T, MT, MST, V6Av, V6Ad, PEci, PGm, 31, 23, 24, PE, PEip, S1, PIVC, OPT, PGop, PG, PFop, Ri, dMIP, VIP, LIP, AIP, 2, 3b, 3a, 1, 46, F1, F3, F7* areas V2, V3, V3A, V4/DP, V4T, MT, MST, V6Av, V6Ad, PEci, PGm, 31, 23, 24, PE, PEip, S1, PIVC, OPT, PGop, PG, PFop, Ri, dMIP, VIP, LIP, AIP, 2, 3b, 3a, 1, 46, F1, F3, F7. Others abbreviations as in Fig. 1

The SPL represents an interface between the visual and somatosensory domains. Accordingly, in the SPL there are cortical areas dominated by the visual input posteriorly, at the border with the occipital pole, and areas dominated by the somatosensory input anteriorly, at the border with the primary somatosensory cortex (Fig. 2A). The cortical region in between shows intermediate functional properties, with a progressive decrease of visual effectiveness moving rostrally that leaves the floor to a parallel increase of somatosensory responsiveness (Fig. 2B). As shown in Fig. 2A, the caudal-most area in SPL is V6 (Galletti et al. 1999a), an extrastriate visual area located in the ventralmost part of the anterior wall and fundus of parieto-occipital sulcus (POs). Area V6 shows a clear occipital cytoarchitectural pattern (Luppino et al. 2005) and belongs to the Brodmann's area 19 (Gamberini et al. 2020). Anterior to V6, three visuomotor areas are located: in the anterior bank of POs (area V6A; Galletti et al. 1999b), subdivided into a ventral—V6Av—and a dorsal—V6Ad—subarea (Luppino et al. 2005; Gamberini et al. 2011), in the medial wall of the intraparietal sulcus (area MIP; Colby et al. 1988), and on the dorsal exposed surface of caudal SPL (area PEc; Pandya and Seltzer 1982) (Fig. 2A). All three visuomotor areas (V6A, MIP, PEc) show a parietal cytoarchitectural pattern (Luppino et al. 2005), belonging to the Brodmann’s area 7 (Gamberini et al. 2020), and are crucial nodes of the above described "reach-to-grasp" network (Fig. 1B). Anterior to them, there is the somatosensory area PE (Fig. 2A) (Pandya and Seltzer 1982), which belongs to the Brodmann’s area 5 (Pandya and Seltzer 1982).

**Fig. 3:**
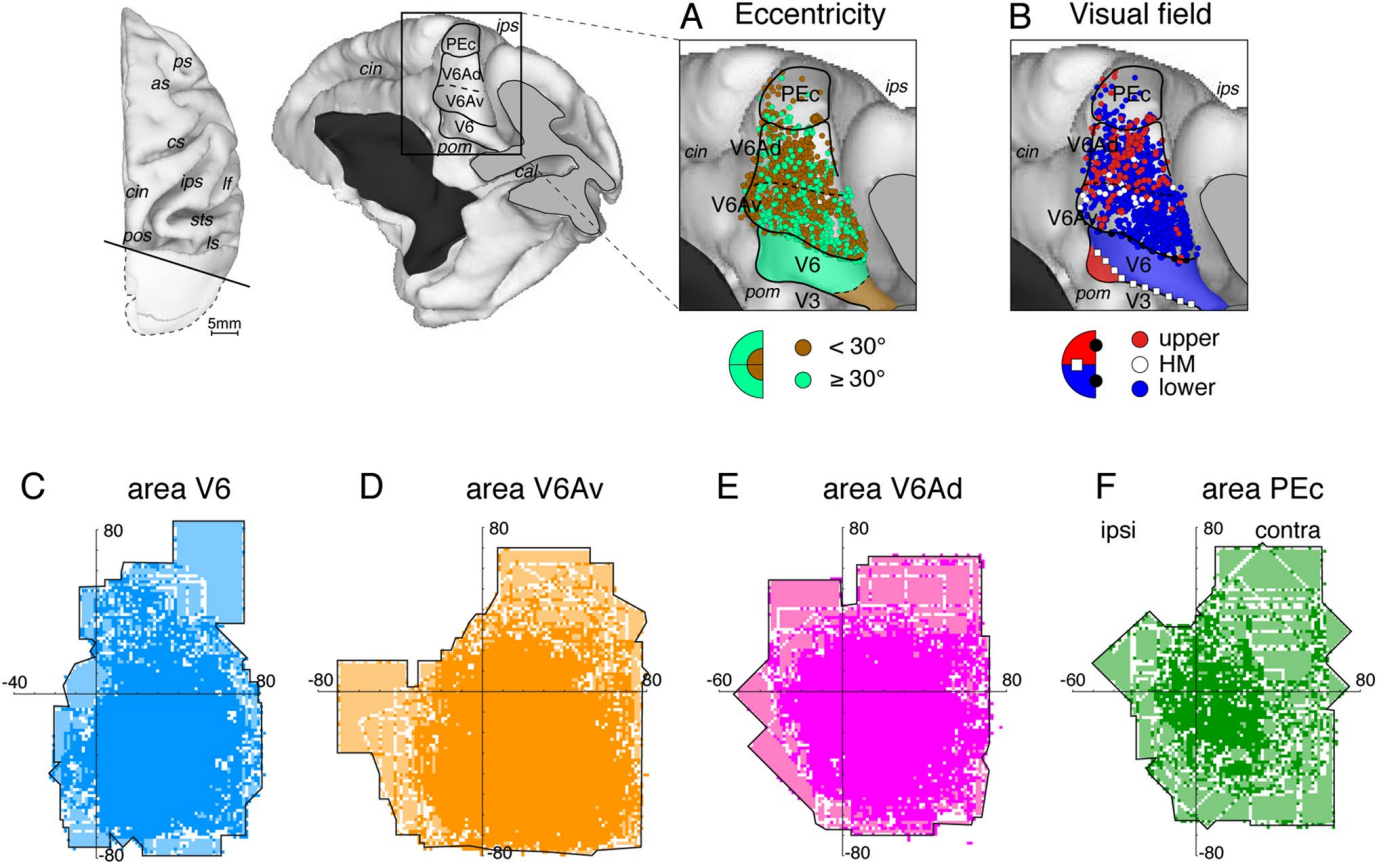
Visuotopic organization and visual field representation in macaque areas V6, V6A and PEc. Dorsal and posteromedial views of a 3D reconstruction of a macaque right hemisphere showing the locations of areas V6, V6Av, and V6Ad in the anterior bank of POs, and the nearby area PEc on the dorsal surface of the SPL. The occipital pole (highlighted in white in the dorsal view) was cut away to show the anterior bank of POs. **A** Distribution in V6A and PEc of visual cells with receptive fields in the central (< 30°; brown dots) and peripheral (> 30°; teal dots) parts of the visual field, respectively. Brown and teal areas in V6 indicate the progression of receptive field eccentricity in the different parts of V6, according to the color coding shown at the bottom. **B** Distribution in V6A and PEc of visual cells with receptive fields in the lower (blue dots) or upper (red dots) visual field. White dots indicate receptive fields located on the horizontal meridian. Blue and red areas in V6 indicate the progression of visual field representation in different parts of V6 according to the color coding shown at the bottom. White squares and black circles represent the HM and VM meridians of area V6. Data obtained from the following studies (Gamberini et al. 2015, 2018). **C**–**F** Distribution of receptive fields (light blue for V6 = 492, orange for V6Av = 585, pink for V6Ad = 324 and green for PEc = 56) with an outline of the most peripheral receptive field borders. The parts of the visual field where the receptive fields are more numerous and superimposed are represented with darker colors. Other abbreviations as in Fig. 1

To reach and grasp an object, it is necessary to localize its spatial location and recognize its visual features (shape, size, orientation), to move the arm (hand) in the right direction and amplitude and to shape the hand according to the features of the object to be grasped (Gottlieb 2007; Vesia and Crawford 2012; Land 2014). In this section, we will summarize the functional properties, and in particular the visual properties, recognized in SPL neurons that are useful in the control of reaching to grasp movements. The functional properties of SPL neurons have been studied in awake non-human primates thanks to hundreds of extracellular microelectrode recordings (e.g.,Kalaska 1996; Galletti et al. 2003; Battaglia-Mayer et al. 2007; Andersen and Cui 2009; McGuire and Sabes 2011; Caminiti et al. 2017; Galletti and Fattori 2018). Many thousands of neurons recorded from the anterior bank of the parieto-occipital sulcus, where areas V6 and V6A are located, have revealed a clear functional difference between these two areas (Galletti et al. 1999a, b; Gamberini et al. 2011, 2015, 2018). V6 hosts a complete and retinotopically organized representation of the contralateral visual field, in particular of the lower visual field, with an overrepresentation of the visual field periphery (Figs. 3A-C) (Galletti et al. 1999a). Area V6A, instead, shows an overrepresentation of the ventral portion of the contralateral visual field, with a larger representation, with respect to V6, of the ipsilateral visual field (Fig. 3D, E) and a poor visuotopic representation, with the central part of the visual field mainly represented dorsally and the periphery ventrally, at the border with V6 (Fig. 3A), and with intermingled upper and lower visual field representations (Fig. 3B) (Galletti et al. 1999b; Gamberini et al. 2011, 2015, 2018). In area PEc, the visual cells are a minority of neuronal population, they are not retinotopically organized (Breveglieri et al. 2008; Gamberini et al. 2018), and most of them represent the central 30° of the contralateral visual field, particularly the lower hemifield (Fig. 3A, B, F), like in area V6Ad. Most visual cells in areas V6, V6A, and PEc are sensitive to the direction of movement of visual stimuli, but since the incidence of visual cells decreases from V6 to PEc, the total number of cells sensitive to the direction of movement decreases accordingly from V6 to PEc. In area MIP, only a few studies have investigated to date the functional properties of single neurons. Colby and Duhamel (1991) reported that in the dorsal part of MIP neurons responded well to passive and active somatosensory stimulation of the limbs, while deeper in the intraparietal sulcus neurons responded well to visual stimulation. In area PE, the visual cells are virtually absent (Duffy and Burchfiel 1971; Mountcastle et al. 1975; Padberg et al. 2007; De Vitis et al. 2019).

The different representation of visual field in SPL areas, together with the functional properties of their neurons, is likely tied to the functional role played by these areas. The retinotopic representation of the whole visual field in V6, including the far periphery, together with the high sensitivity of its neurons to the orientation, size, and direction of movement (Galletti et al. 1996), and the ability of many of them to recognize the real movement of objects (real-motion cells; Galletti and Fattori 2003), are all properties well suited for inferring specific properties of the objects to be grasped and for grasping them correctly, particularly when the objects are in motion in the visual field. These properties are particularly useful when we are looking around while advancing through a structured environment, that is, when a lot of images move upon the retina, partly as a consequence of self-motion and partly evoked by real movement of objects in the visual world. The real-motion cells recognize the objects that really move in the visual field, everywhere in the field of view. We suggest that V6 provides this type of visual information to the visuomotor centers involved in the control of reaching to grasp movements, like areas V6A and PEc (Galletti and Fattori 2018; Gamberini et al. 2020). To this regard, it is worthwhile to notice that in V6, V6A, and PEc, the lower quadrant of the visual field is overrepresented (Fig. 3C–F). This part being the peripersonal space usually passed through by the limbs during reaching to grasp a foveated object (Fattori et al. 2017) and also the part of peripersonal space where we look at or attend to during locomotion to avoid obstacles, the overrepresentation of the lower hemifield is a property useful to guide and control goal-directed limb movements (Fattori et al. 2017; Galletti and Fattori 2018).

In addition to be involved in real-motion detection and in providing visual information for controlling reaching and grasping, V6 could also provide useful visual information to other cortical areas involved in the control of locomotion and navigation. Indeed, V6 represents the whole visual field including the far periphery (Galletti et al. 1999a; Fattori et al. 2009), hosts plenty of direction selective and real-motion cells (Galletti and Fattori 2003), and is activated by optic flow mimicking self-motion through a structured environment (Fan et al. 2015; Pitzalis et al. 2021) (but see Cottereau et al. 2017), all visual features important for navigation and visual guidance of locomotion. Several recent neuroimaging studies in humans support this view. It has been found, for instance, that the human homolog of macaque V6 represents the whole visual field including the far periphery (Pitzalis et al. 2006, 2015), is highly selective to the direction of movement (Pitzalis et al. 2006, 2010), to the real motion (Fischer et al. 2012a, b; Nau et al. 2018), and to the optic flow stimulation mimicking self-motion (Cardin and Smith 2010, 2011; Pitzalis et al. 2010, 2013; Di Marco et al. 2021). In addition, human V6 responds more to scenes/places compared to faces (Sulpizio et al. 2020), another feature that could help in navigation and in guidance of self-motion.

**Fig. 4:**
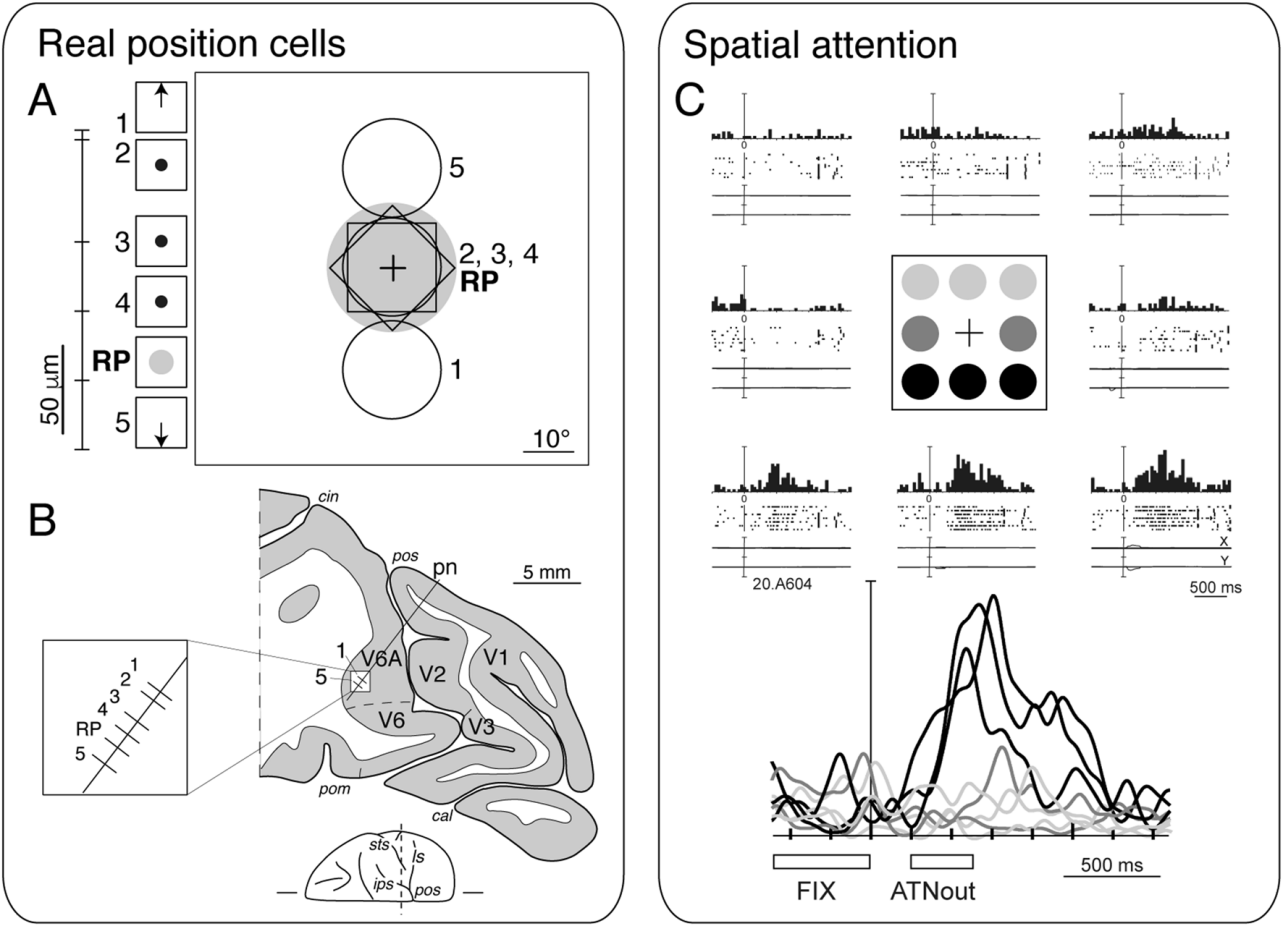
Details of specific functional properties in area V6A. **A** Receptive field locations and preferred gaze directions of a cluster of cells recorded at different depths in area V6A, as indicated in (**B**), according to the scale reported on the left. The gray area indicates the screen location of the visually responsive region of the real-position cell. RP indicates a real-position cell. In the small squares, beside cell numbers on the left, the direction of the gaze modulation of visual responsiveness is reported: upward and downward arrows indicate that cells were visually responsive only when the animal looked upward and downward, respectively; point at the center indicates that cells were visually responsive only when the animal looked at the center of the screen. **B** Reconstruction of a microelectrode penetration through area V6A. Numbers 1–5 along the electrode track (pn) indicate the locations of the cluster of neurons grouped around a real-position cell. **C** Example of spatially tuned modulations of neural activity during outward attention epoch. Each inset contains the perievent time histogram, raster plots and eye position signals, and is positioned in the same relative position as the cue on the panel. In the bottom part of the figure, the spike density functions (SDFs) of the activity for each of the eight cue positions are superimposed and aligned on the cue onset. The mean duration of epochs FIX and outward attention is indicated below the SDFs. Neural activity and eye traces are aligned on the cue onset. Scale bar in perievent time histograms, 70 spikes/s. Bin width, 40 ms. Eye traces: scale bar, 60°. Data obtained from the following studies (Galletti et al. 1995, 2010)

In area V6A, the visual cells are not retinotopically organized as in V6. Neurons with receptive field located in different parts of the visual field are one near to the other, so the visuotopic organization of the area is severely ‘blurred’. It has been suggested that this apparently chaotic visuotopic organization is necessary to build up the so-called ‘real-position’ cells, that is cells whose receptive field remains constant in space regardless of eye position and movement (Galletti et al. 1993). Indeed, real-position cells are present in V6A, intermingled with gaze-dependent visual neurons with receptive field located in different parts of the visual field (Fig. 4A, B), embedded in functional modules well suited to encode the spatial locations of objects in the visual field (see Galletti et al. 1995). V6A neurons are also modulated by the shift of spatial attention and it has been suggested that the spatial coordinates encoded by real-position cells could be used to direct the spotlight of attention towards the attended object (Galletti et al. 2010) (Fig. 4C). The presence in V6A of cells modulated by the direction of gaze (Galletti et al. 1995; Hadjidimitrakis et al. 2011; Breveglieri et al. 2012) and by the direction and amplitude of goal-directed arm movements (Fattori et al. 2005; Hadjidimitrakis et al. 2014), as well as of cells modulated by the shape of hand according to the grasped object (Fattori et al. 2012), well agree with the view that V6A is directly involved in the control of reaching to grasp actions. Recently, it has been shown that individual cells in V6A are modulated by most of the above recalled factors, showing mixed selectivity (Diomedi et al. 2020). This mixed selectivity that builds up a dynamic representation of visuospatial and visuomotor information has been demonstrated to be computationally efficient (Fusi et al. 2016) and less prone to errors than pure selectivity (Johnston et al. 2020). The tuning of cell activity to each factor is not static, but changes with time, indicating the sequential occurrence of visuospatial and visuomotor transformations occurring in V6A, a behavior helpful to guide a goal-directed arm movement (Hadjidimitrakis et al. 2017; Diomedi et al. 2020).

In area PEc, visual neurons show functional properties quite similar to those of area V6A (Breveglieri et al. 2008), the only difference being their incidence in the total cell population. While in V6A visual cells represent about 60% of the total, in PEc they represent 40% of the total cell population (Gamberini et al. 2018). The remaining 60% of PEc neurons are somatosensory or somatomotor in nature, like about 40% of V6A cells (Breveglieri et al. 2006). However, somatosensory and somatomotor neurons show remarkable differences in the two areas. In both areas the arms are overrepresented, but while V6A represents only the upper limbs, area PEc represents both the upper and lower limbs (Gamberini et al. 2018). We therefore suggested that V6A is involved in the control of object prehension performed with the upper limbs, while PEc in the control of hand/foot interaction with the objects of the environment and in locomotion (Gamberini et al. 2018). A recent neuroimaging study in humans confirms this suggestion for the homologous areas of the human brain, showing in particular that the putative human homolog of PEc responds to both arm and leg movements and to flow field visual stimulation similarly to macaque area PEc (Pitzalis et al. 2019).

As recalled above, in area PE the visual cells are virtually absent and most of the neurons respond to proprioceptive stimulation (Duffy and Burchfiel 1971; Mountcastle et al. 1975; Padberg et al. 2007). This holds true not only for the most lateral parts of PE, the usual target of neurophysiological investigations, but also for the most medial part of the area, where strong somatosensory/somatomotor responses, but no visual cells, have been recently found (De Vitis et al. 2019). In PE it has been recognized a rough topographic representation of the body, dominated by the representation of the upper limbs while the legs are less represented (Padberg et al. 2007; Seelke et al. 2012). Considering the main topic of this work, centered on the visual input to SPL, area PE will be here treated only for comparison with the other SPL areas.

## Cortical connections of macaque SPL

In this section, we will describe the cortical inputs to SPL based on injections of retrograde neuronal tracers in separate areas of this structure, taking into account the recent areal subdivision of SPL (Fig. 2A) (Gamberini et al. 2020).

The visual area V6 shows direct cortical afferents that originate from the striate area V1 as well as from many extrastriate visual areas of the occipital lobe (Fig. 2C, blue lines). This visual input represents more than 70% of the total cortical input to area V6 (Galletti et al. 2001), which also receives afferents from bimodal, somatovisual parietal areas located in the parieto-occipital and intraparietal sulci (less than 30% of labeling). No labeled cells were found in the inferior temporal, mesial and frontal cortices.

**Fig. 5:**
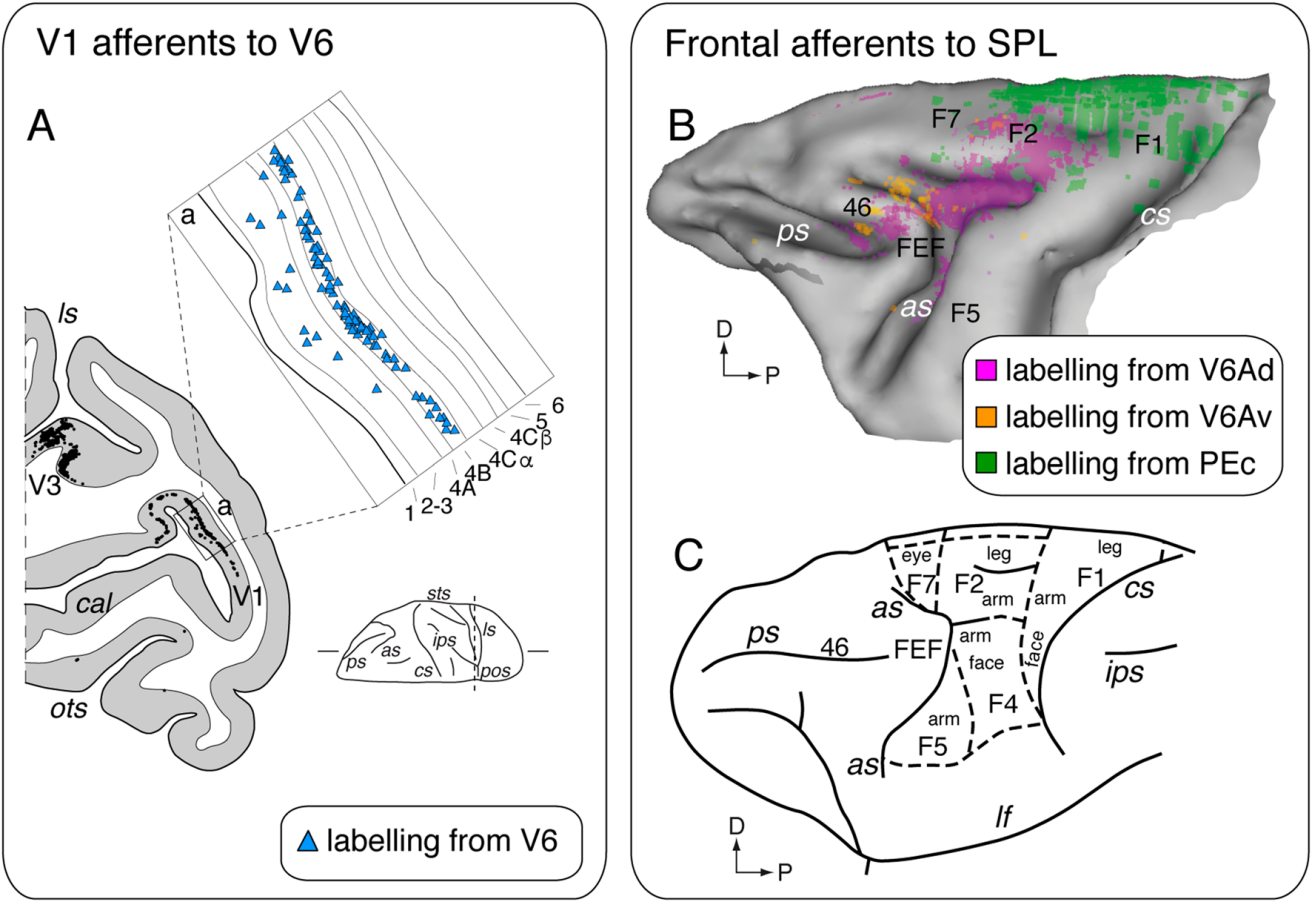
Details of specific cortical connections. **A** Laminar pattern of labeling in V1 after V6 tracer injection. Caudal part of a parasagittal section taken at the level indicated on the brain silhouette at the bottom-right. Each single black dot represents a retrogradely labeled cell. The inset 'a' showing an enlargement of a part of the posterior branch of calcarine fissure (squared area on the section). Light blue triangles are single retrogradely labeled cells. Numbers and letters indicate the cortical layers in V1. **B** Frontal connections of areas PEc, V6Av, and V6Ad. Frontal lobe with cells (colored dots) labeled after retrograde tracer injection in areas PEc (green; three injections), V6Av (orange; two injections), and V6Ad (pink; three injections). Data obtained from Gamberini et al. (2009); Bakola et al. (2010); Passarelli et al. (2011) **C** Parcellation of agranular frontal cortex showing, overimposed, the representations of various body parts. Data modified from the following studies: Matelli et al. (1991); Luppino and Rizzolatti (2000). Other details and abbreviations as in Figs. 1 and 2

According to the retinotopic organization of V6 (Fig. 3), injections of retrograde neuronal tracer in its central or peripheral representation produced a strong labeling in central or peripheral representation, respectively, of area V1. Labeled cells in V1 were mainly concentrated in the layer IVB and less evident in supragranular layers 2 and 3 (Fig. 5A). Layer IVB belongs to the magnocellular pathway (Lund et al. 1975), where most of the cells are tuned for orientation and direction of motion of visual stimuli (Dow 1974; Zeki 1978). Both selectivities are well represented also in area V6, as described in the previous section. As illustrated in Fig. 2C, the extrastriate visual areas connected with V6 were MT/V5, V3, V3A and, less strongly, V2, V4T, and V4/DP. All these connections respected the visuotopic organization of the areas. Note that the high majority of V6 afferents (blue lines in Fig. 2C) are in the occipital cortex and the region of the superior temporal sulcus. Area V6 is also connected to the ventral part of areas V6A (V6Av) (Galletti et al. 2001; Passarelli et al. 2011), to area MIP (Galletti et al. 2001; Bakola et al. 2017), LIPv, and VIP (Colby et al. 1988; Baizer et al. 1991; Galletti et al. 2001). Since V6 hosts only visual neurons (Galletti et al. 1999a; Gamberini et al. 2015) and does not receive any input from pure somatic regions of the brain (Galletti et al. 2001), we suggest that the type of input V6 receives from the bimodal parietal areas V6A, MIP, LIP and VIP is visual in nature. According to this view, labeled cells in V6A, MIP and LIP after V6 injection were confined in the ventral part of these areas, where previous functional experiments have demonstrated that cells were more likely activated by visual stimulations (Blatt et al. 1990; Colby and Duhamel 1991; Gamberini et al. 2011).

We found that the distribution of labeling within areas VIP and LIPv did not depend on central or peripheral injections in V6, suggesting that both parietal areas are not retinotopically organized (Galletti et al. 2001). In literature, this is a contrasted result. Some functional experiments too reported that VIP is not retinotopically organized (Colby and Duhamel 1991; Colby et al. 1993), but anatomical experiments revealed segregated afferents to LIP and VIP after injections in different parts of retinotopically organized areas (Colby et al. 1988; Andersen et al. 1990; Baizer et al. 1991), suggesting for these areas an at least coarse visuotopic organization. Other functional studies showed that the dorsal part of LIP is dominated by the representation of the central part of the visual field and the ventral part of LIP, the only one connected with V6, is dominated by the representation of the periphery (Blatt et al. 1990; Hamed et al. 2001; Arcaro et al. 2011). Interestingly, this result mimics the V6–V6A connections, where only the ventral part of V6A, representing the far periphery of the visual field, receives afferents from V6 while the dorsal part of V6A, representing mostly the central part of the visual field, is not directly connected with V6 (Galletti et al. 2001). The other bimodal SPL area connected with V6 is area MIP. Peripheral V6 injections produced labeling in the ventral portion of MIP, while central injections produced labeling in a dorsal portion of it (Galletti et al. 2001), suggesting a rough topographical organization for this area. In summary, all bimodal areas V6A, MIP, LIP, and VIP are connected with both central and peripheral representation of V6, but in areas V6A and MIP labeled cells were found in different regions whereas in LIP and VIP they were found in about the same cortical region. In other words, our connectional data suggest an at least coarse retinotopic organization for V6A and MIP, the former in agreement with previous functional experiments and the latter not yet investigated to this regard, and a non-retinotopic organization for LIP and VIP, the former of which is in contrast to the functional and connectional data reported in literature. It could also be that the connections of V6 with these areas are dictated by rules other than the visuotopic ones, rules that strictly depend on the functional role these areas play. Further functional and anatomical experiments are needed to clarify this point.

As recalled above, the area V6A hosts visual and somatosensory neurons, both types of cells non-topographically organized (Gamberini et al. 2011). The ventral part of area V6A (V6Av; Fig. 2) receives visual information (about 60% of total labeling) from the extrastriate areas of the occipital and temporal lobe (V2, V3, V4, MST, V6), but not from V1 (Fig. 2C, orange lines). Area V6Av is strongly and reciprocally connected with the dorsal part of V6A (V6Ad; Fig. 2) (Gamberini et al. 2009; Passarelli et al. 2011) and with area MIP (Passarelli et al. 2011; Bakola et al. 2017). Both V6A and MIP being bimodal areas, it is likely that visual information runs from V6Av to V6Ad and MIP and, conversely, somatosensory information from V6Ad and MIP to V6Av. Both V6Av and V6Ad (Fig. 2C, orange and pink lines) are connected with the other bimodal areas of the posterior parietal cortex (37% of total labeling V6Av; 78% V6Ad). The principal sources of bimodal input are the SPL areas MIP, PEc, and PGm, followed by the intraparietal areas AIP, LIP, and VIP, and the IPL area PG. Notice that the somatosensory inputs to V6A could arise from the bimodal parietal areas but not from the somatosensory cortex, because this latter was found not directly connected with V6A (Fig. 2C) (Gamberini et al. 2009; Bakola et al. 2010, 2017; Passarelli et al. 2011). Area V6Ad, but not V6Av, receives afferents from the frontal lobe (14% of total afferences), in particular from the dorsal premotor areas F2 and F7 and, more weakly, from area 46.

Anterior to V6A, on the convexity of SPL, there is area PEc (Fig. 2A), a parietal bimodal area hosting visual and somatosensory neurons like area V6A, but with a smaller incidence of visual cells and a higher incidence of somatosensory cells (Gamberini et al. 2018). Like in area V6A, also in PEc both visual and somatosensory neurons are not topographically organized (Gamberini et al. 2018). As shown in green in Fig. 2C, PEc is strongly connected with areas V6A and MIP, in particular V6Ad and dMIP, that is, the part of the two areas more abundant in somatosensory neurons (Colby and Duhamel 1991; Gamberini et al. 2011). As recalled above, both V6A and MIP are bimodal areas like PEc. It is therefore likely that these areas exchange both types of sensory information, visual and somatic, though we have no tool to determine whether and where one possibly prevails over the other. PEc also receives strong afferents from inferior parietal (PG, PGop) and mesial (23, 24, 31, PEci) areas. The PEci is a somatosensory area (also called supplementary somatosensory area or SSA; Murray and Coulter 1981) therefore, it is likely that it exchanges with PEc somatosensory information. Weak somatosensory inputs arise also from areas 2 and PE (about 4% of total connections). Finally, area PEc is well connected with the frontal lobe (about 17% of labeling), mainly with the premotor area F2. Interestingly, the F2 afferents to PEc are complementary to those to V6A, in that PEc mainly receives input from the portion of F2 representing the lower limb, while V6A receives input almost exclusively from the premotor region representing the upper limb (Fig. 5B and C). This matches with the functional properties of these two areas, with V6A representing almost exclusively the upper limb and PEc representing both upper and lower limb.

As shown in Fig. 2A, area PE is located in the most anterior part of SPL and does not receive any visual input (Fig. 2B). Figure 2C (yellow lines) shows that PE receives strong somatosensory input from the primary somatosensory cortex, in particular area 2, and from some areas of the parietal cortex of both superior and the inferior parietal lobules. PE also receives strong motor afferents from the primary motor area F1 and the premotor area F3 (Bakola et al. 2013).

Notice that overall the afferents to SPL are in strict agreement with the functional gradient observed in this structure, with the caudalmost part dominated by visual properties and visual afferents, and the most anterior part dominated by somatosensory properties and afferents.

## Thalamic connections of macaque SPL

It has been known for decades that the macaque SPL receives a strong thalamic input from the pulvinar complex and the lateral posterior nucleus, as well as a weaker input from several other thalamic nuclei (Yeterian and Pandya 1985, 1997; Schmahmann and Pandya 1990; Grieve et al. 2000). In the light of a recent description of areal subdivision in the SPL (see Fig. 2A), we will describe in the following the specific thalamic afferents to the SPL areas V6, V6A, PEc, and PE.

**Fig. 6:**
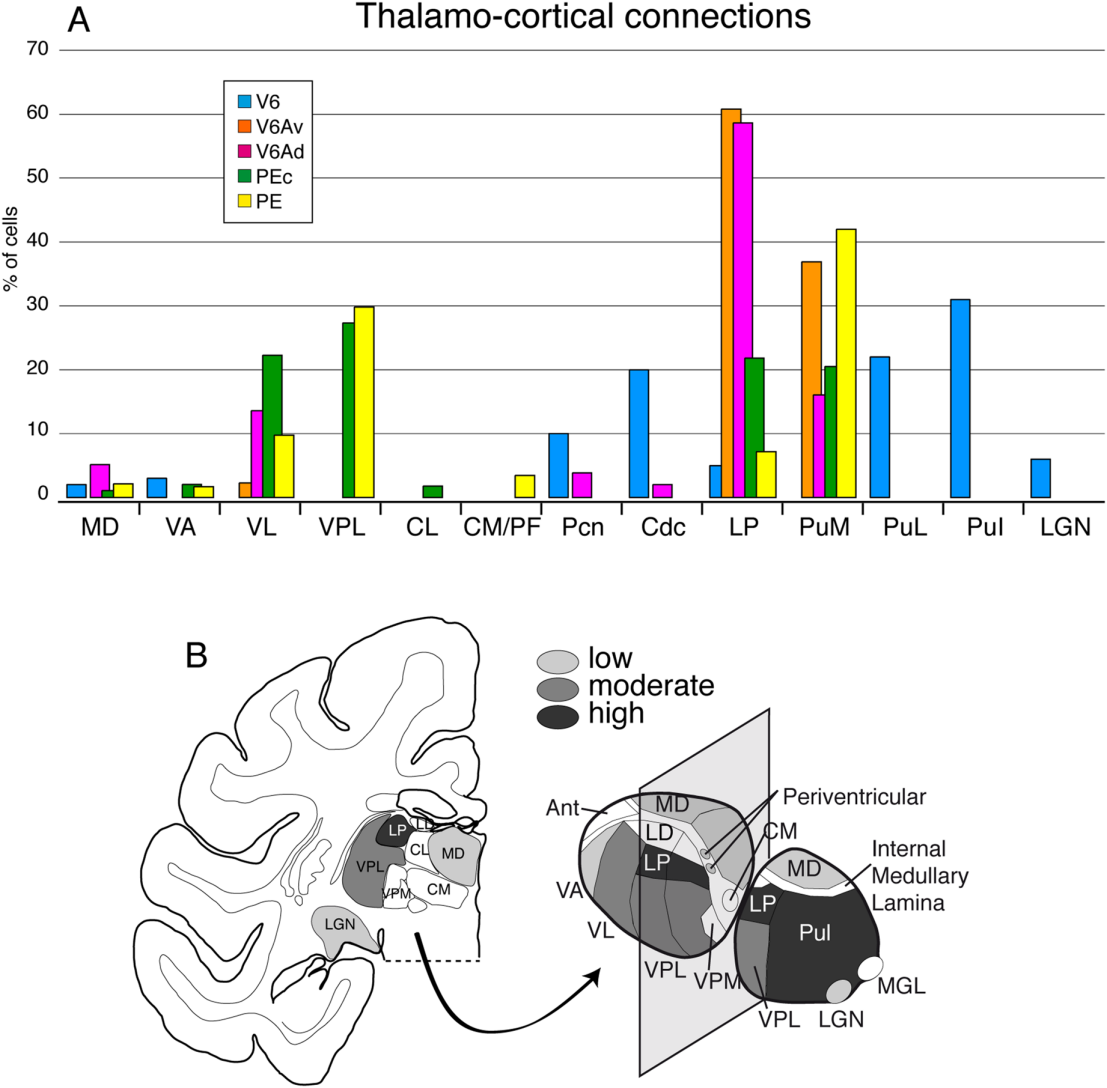
Thalamocortical connections of the SPL areas. **A** Average percentages of labeled cells in thalamic nuclei after tracer injections in areas V6, V6Av, V6Ad, PEc, and PE. Only labeling that represented > 1% of the thalamic afferents are reported. **B** Coronal section, left, taken approximately at the center of the thalamus shown on the right. Schematic representation of the thalamus with continuous lines delimiting the different thalamic nuclei: nuclei highlighted with different shades of gray are highly (more than 30% of thalamic afferents), moderately (> 5 < 30% of thalamic afferents), or weakly (less than 5% of thalamic afferents) connected with SPL areas. Data obtained from Gamberini et al. (2016) and Impieri et al. (2018). *MD* medial dorsal, *VA* ventral anterior, *VL* ventral lateral, *VPL* ventral posterior lateral, *VPM* ventral posterior medial, *CL* central lateral, *CM/PF* centromedian/parafascicular, *Pcn* paracentral, Cdc central densocellular, *LD* lateral dorsal, *LP* lateral posterior, PuM medial subdivision of pulvinar, *PuL* lateral subdivision of pulvinar, *PuI* inferior subdivision of pulvinar, *LGN* lateral geniculate nucleus, *MGL* medial geniculate nucleus. Other details and abbreviations as for Figs. 1 and 2

As shown in Fig. 6A (light blue columns), the major thalamic projections to area V6 (about 60% of labeling) arise from pure visual nuclei, in particular the lateral (PuL) and inferior (PuI) portions of the pulvinar complex (Gamberini et al. 2016). These results are in good agreement with a previous report (Shipp et al. 1998). A minor visual projection to V6 also comes from the lateral geniculate nucleus (LGN), specifically from the interlaminar layers of this nucleus. The distribution of labeled cells we found in LGN followed the topography of the visual field represented in this structure, thus the central representation in V6 receives from the central representation in LGN and the periphery in V6 from the peripheral representation in LGN. While peripheral injections in V6 showed afferents limited to PuL, PuI and LGN, after central injections labeled cells were also found in mediodorsal (MD), intralaminar (Pcn nucleus) and periventricular (CdC nucleus) nuclei. These nuclei strongly contribute to the thalamic afferents to SPL, representing more than 30% of the total input, but their specific functions are still not completely understood. However, according to the few studies focused on their functional properties (Huerta and Kaas 1990; Watanabe and Funahashi 2004; Hsu and Price 2007; Hsu et al. 2014), this thalamic input would bring to V6 visual and oculomotor related signals that matched the functional properties typical of area V6 (see the above section on the functional properties of V6).

The major thalamic afferents to V6A come from LP and medial pulvinar (PuM) nuclei (Gamberini et al. 2016). In particular, V6Av receives about 98% of its thalamic afferents from these two nuclei and V6Ad about 75% (Fig. 6A, orange and pink columns, respectively). Since LP and PuM are multimodal associative nuclei (Ma et al. 1999; Kamishina et al. 2008, 2009), these thalamic inputs likely bring to V6A both visual and somatic information. These types of inputs well agree with the integrative, visuomotor role of area V6A described above. Minor thalamic motor inputs to V6A come from the ventrolateral nucleus (VL) (Ilinsky and Kultas-Ilinsky 1987), that should bring somatomotor information (Vitek et al. 1994; Mai and Forutan 2012). The majority of VL input (about 14%) reaches V6Ad and a minority (about 2%) V6Av. The different incidence of visual, somatosensory, and somatomotor thalamic inputs to the two cortical areas mimics their functional differences, with V6Av being more visual and less somatomotor than V6Ad. Differently to the cortical afferents, where V6Av receives direct visual information from the extrastriate area V6, both sectors of V6A do not receive pure visual thalamic inputs. V6Ad, but not V6Av, receives also afferents from MD, which is a thalamic nucleus known to be involved in the control of the direction of gaze and the spotlight of attention (Schlag and Schlag-Rey 1984; Schlag-Rey and Schlag 1984; Watanabe and Funahashi 2004). As described above, area V6A takes part in the guidance of intentional motor acts (Galletti et al. 2003; Gamberini et al. 2011; Fattori et al. 2017) and has been reported to be influenced by the shift of spatial attention, both in monkeys (Galletti et al. 2010; Caspari et al. 2015) and humans (Ciavarro et al. 2013; Caspari et al. 2018). Interestingly, these functional properties well agree with the thalamic inputs described above. Additional weak thalamic afferents to V6Ad, but not to V6Av, arise from Pcn and CdC nuclei that provide visual and oculomotor inputs to the cortex (Huerta and Kaas 1990; Hsu and Price 2007; Hsu et al. 2014). Surprisingly, V6Ad mainly represents the central 30° of the visual field (see Fig. 3A) and these same thalamic nuclei (Pcn and CdC) also project to the central representation of area V6 (see Fig. 6A). These common thalamic afferents of central V6 and V6Ad seem to be useful for the functional roles played by the two cortical areas. Indeed, we suggested that visual and gaze signals are used by these cortical areas to foveate visual targets and to control arm/hand movements to reach and grasp those targets (Gamberini et al. 2011; Fattori et al. 2017; Galletti and Fattori 2018). Area V6Av that receives strong visual input from peripheral V6 and projects to V6Ad could be involved in bringing on the fovea the image of objects located in the periphery of the visual field, to allow a direct visual control of grasping movements. Interestingly, the cells encoding location in space regardless of gaze direction (the real-position cells; Galletti et al. 1993) are segregated in V6Av (Galletti et al. 1999b). The output of real-position cells could reach V6Ad to guide the shift of the spotlight of attention (and the shift of gaze) toward the objects of interest. According to this hypothesis, it has been reported that V6A is strongly activated during the shifts of spatial attention (Caspari et al. 2015). A similar activation has been recently reported also in the homologous brain region in humans (Caspari et al. 2018).

Thalamic inputs to areas PEc and PE mostly originate from LP and PuM (42% of total thalamic connections to PEc and 49% to PE), from the ventral posterior lateral nucleus (VPL) (27% to PEc and 30% to PE), and from VL (22% to PEc and 10% to PE) (Impieri et al. 2018) (Fig. 6A, green and yellow columns, respectively). As reported above, the LP and PuM inputs should bring associative visuosomatic information (Ma et al. 1999; Kamishina et al. 2008, 2009), VPL somatosensory information (Rausell et al. 1998), and VL somatomotor information (Vitek et al. 1994; Mai and Forutan 2012). Surprisingly, these thalamic inputs do not mimic the functional properties of the two SPL areas, with PEc being a bimodal, visuosomatic area and PE an almost pure somatic one. However, we actually do not know whether the associative visuosomatic nuclei of the thalamus project to PEc and PE the same type of input, visual, somatic, or mixed. A possibility is that the thalamic input to PE is only somatic in nature. Alternatively, PE neurons may be modulated also by visual stimulations not yet tested to date. Future experiments will hopefully clarify this point. Finally, after PEc and PE tracer injections, weak labeling (around 4%) was found in MD and VA, two nuclei involved in the motor control (Schlag and Schlag-Rey 1984; Schlag-Rey and Schlag 1984; Mushiake and Strick 1995; Middleton and Strick 2000; Sommer 2003; Watanabe and Funahashi 2004). This thalamic input seems to be in line with the somatomotor nature of both these parietal areas.

In summary, all the five SPL areas here taken into account receive thalamic afferents from LP and pulvinar complex, with some peculiar specificity. As shown in Fig. 6A, LP sends strong projections to V6A and weaker inputs to V6, PE and PEc; PuM sends strong inputs to all SPL areas but V6; PuI/PuL sends strong projections to V6 but not to the other areas of SPL. As summarized in Fig. 6B, LP and pulvinar complex afferents together represent the strongest thalamic input to SPL. Moderate input to SPL arises from VPL and VL nuclei: VPL sends strong projections to PE and PEc; VL to V6A, PE, and PEc. Neither VPL nor VL send thalamic input to V6. Very weak connections to SPL areas arise from MD, VA, intralaminar nuclei and LGN.

## Conclusions

**Fig. 7:**
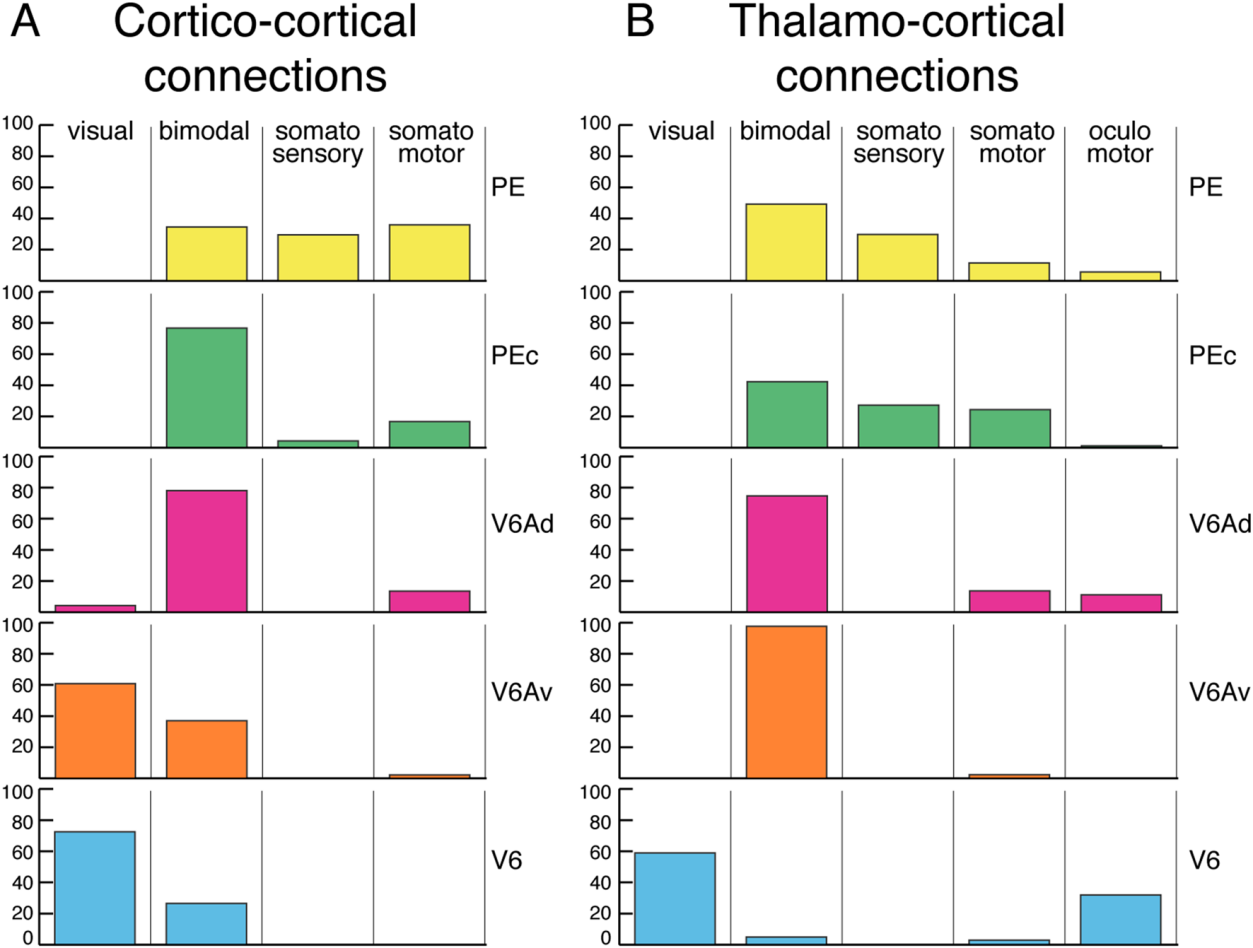
Patterns of cortical and thalamic input to SPL areas. **A** The pattern of cortical connections to SPL areas includes: "visual", striate and extrastriate visual areas; "bimodal", parietal areas located in the superior and inferior parietal lobule and on the mesial surface of the hemisphere; "somatosensory", somatosensory primary and secondary areas as well as multimodal areas in the insular cortex; "somatomotor", frontal, premotor and prefrontal cortex. **B** The pattern of thalamic afferents to SPL areas includes: "visual", the lateral and inferior subdivisions of pulvinar nucleus together with the lateral geniculate nucleus; "bimodal", the lateral posterior and the medial portion of the pulvinar nucleus; "somatosensory", the ventral posterior lateral nucleus; "somatomotor", the ventral lateral and ventral anterior nuclei; "oculomotor", medial nucleus together with intralaminar and periventricular nuclei with oculomotor related activity

The SPL areas receive sensorimotor afferents that are useful for their functional properties. A comparison of Fig. 2B with Fig. 7 is impressive to this regard. Figure 2B summarizes the sensory properties of SPL areas, and Fig. 7 the functional properties of cortical and thalamic afferents to these areas. The afferents in Fig. 7 are grouped according to the functional properties of neurons in the cortical or thalamic region of origin. Five functional categories are used to classify the inputs: ‘visual’, ‘bimodal’, ‘somatosensory’, ‘somatomotor’, and ‘oculomotor’. There is clearly a functional trend in the SPL from the visual input posteriorly, in V6 (Brodmann’s area 19), to the somatosensory/somatomotor input anteriorly, in PE (Brodmann’s area 5). Notice, however, that the ‘pure’ visual area V6 does not receive only visual inputs, but also inputs from bimodal cortical areas and thalamic nuclei. Similarly, the somatosensory area PE receives afferents from both somatosensory and bimodal cortical areas and thalamic nuclei. Why the visual areas receive also somatosensory inputs and the somatosensory areas also visual inputs is at present unknown. It could be that only the visual neurons from the bimodal regions send projection to V6 and, similarly, that only somatosensory inputs reach area PE from the bimodal regions, but currently we have no tools to verify this hypothesis. An issue apart regards the oculomotor input. Many cortical areas projecting to SPL host neurons influenced by the direction of gaze, or by saccades, but it was very difficult to quantify these effects from the data reported in literature, so they are not reported in Fig. 7A. In spite of this, it was clear from literature that the gaze effect is more important in visual and bimodal areas than in somatosensory and somatomotor ones. About the thalamic oculomotor input, it was easier to extract its incidence from the literature, since for some thalamic nuclei oculomotor activity has been specifically reported. The thalamic input related to the gaze effect, for instance, is high in the visual areas of SPL, particularly in area V6, as shown in Fig. 7B. Figure 7 also shows that the SPL areas V6Av, V6Ad, and PEc, which according to Gamberini and co-authors (2020) belong to the ‘associative’ Brodmann’s area 7, receive strong afferents from bimodal cortical and thalamic regions and represent the most integrative part of the SPL, where information about the body state (in particular limb position) and visual environment merged together to allow and refine reach-to-grasp actions and locomotion (Galletti and Fattori 2018; Gamberini et al. 2020).

**Fig. 8:**
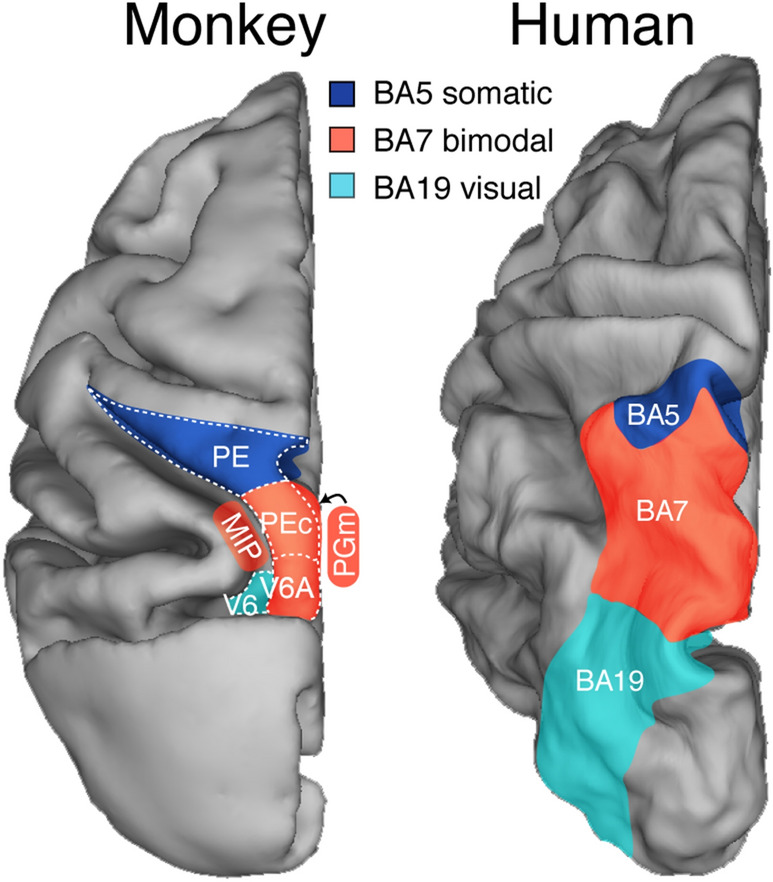
Comparison between monkey and human SPLs. Left, dorsal view of the left hemisphere of macaque brain showing the location and extent of a number of SPL areas: the region colored in light blue is the visual cortex (that includes area V6) and belongs to Brodmann’s area 19; the region colored in orange, which includes areas PEc, V6A, MIP, and PGm, is responsive to both visual and somatosensory stimulations and belongs to Brodmann’s area 7; the region colored in blue (that includes area PE) is responsive to somatosensory stimulation but not to visual stimulation, and belongs to Brodmann’s area 5. Right, dorsal view of the left hemisphere of the human brain showing the location and extent of Brodmann’s areas 5 (blue), 7 (orange), and 19 (light blue). Modified from Gamberini et al. (2020)

About possible homologies between macaque and human SPL, it is a common view that they are not homologous structures, because the macaque SPL is almost completely occupied by Brodmann’s area 5, whereas the human SPL is mainly occupied by area 7 (Fig. 8). Recently, a different interpretation has been proposed by Gamberini and coworkers (Gamberini et al. 2020). Since Brodmann (1909) did not specify the extent of areas in the depth of brain sulci, and since based on functional and anatomical criteria (see Gamberini et al. 2020) the cortical regions hidden in the parieto-occipital and intraparietal sulci (areas V6A and MIP) likely belong to area 7, the hypothesis was advanced that area 7 in macaque monkey is larger than previously indicated, including areas V6A, MIP, PEc, and PGm (Fig. 8). According to this hypothesis, the human and monkey SPL would be organized in a similar way (as shown in Fig. 8), with a large area 7 posteriorly and a smaller area 5 anteriorly. Also the functional comparison between macaque and human SPL as reviewed in the present work seems to strongly support this similarity.

The unpredicted anatomical and functional similarity between macaque and human SPL could have important relapses. It could help, for instance, in understanding the controversial neurological origin of the optic ataxia disease. In optic ataxia patients, in fact, the caudal aspect of SPL is typically affected by brain damage (Perenin and Vighetto 1988; Karnath and Perenin 2005). As reviewed here, caudal SPL neurons encode the location in space of objects, direction of arm movement, and shaping of the hand during grasping. It is therefore logical that a lesion of this part of the brain produces reaching and grasping impairments, as typically observed in optic ataxia patients. Furthermore, the knowledge of functional properties and anatomical circuits that characterize the SPL of primates, together with the view that this structure has the same anatomical and functional organization in human and non-human primates, could allow to build up an artificial interface able to guide human neuroprosthetic devices acting in dynamic environments in a natural, smooth, and fast way.

## Data Availability

Not applicable.
